# Effective connectivity between bed nucleus of the stria terminalis and amygdala: Reproducibility and relation to anxiety

**DOI:** 10.1002/hbm.25265

**Published:** 2020-11-06

**Authors:** David Hofmann, Thomas Straube

**Affiliations:** ^1^ University Hospital Muenster Institute of Medical Psychology and Systems Neuroscience Muenster Germany

**Keywords:** amygdala, anxiety, bed nucleus of the stria terminalis, dynamic causal modeling, effective connectivity, resting‐state fMRI

## Abstract

In a previous study, we investigated the resting‐state fMRI effective connectivity (EC) between the bed nucleus of the stria terminalis (BNST) and the laterobasal (LB), centromedial (CM), and superficial (SF) amygdala. We found strong negative EC from all amygdala nuclei to the BNST, while the BNST showed positive EC to the amygdala. However, the validity of these findings remains unclear, since a reproduction in different samples has not been done. Moreover, the association of EC with measures of anxiety offers deeper insight, due to the known role of the BNST and amygdala in fear and anxiety. Here, we aimed to reproduce our previous results in three additional samples. We used spectral Dynamic Causal Modeling to estimate the EC between the BNST, the LB, CM, and SF, and its association with two measures of self‐reported anxiety. Our results revealed consistency over samples with regard to the negative EC from the amygdala nuclei to the BNST, while the positive EC from BNST to the amygdala was also found, but weaker and more heterogenic. Moreover, we found the BNST‐BNST EC showing a positive and the CM‐BNST EC, showing a negative association with anxiety. Our study suggests a reproducible pattern of negative EC from the amygdala to the BNST along with weaker positive EC from the BNST to the amygdala. Moreover, less BNST self‐inhibition and more inhibitory influence from the CM to the BNST seems to be a pattern of EC that is related to higher anxiety.

## Graphical abstract

We investigated the reproducibility of resting‐state fMRI effective connectivity (EC) between the bed nucleus of the stria terminalis (BNST) and amygdala nuclei and its relation to anxiety. We found a reproducible pattern of negative EC from the amygdala to the BNST and a positive EC from the BNST to the amygdala. Less BNST self‐inhibition and higher inhibitory influence from the CM to the BNST was associated with higher anxiety.

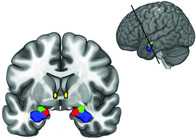

## 1 INTRODUCTION

Within the complex network of highly interacting brain regions, the bed nucleus of the stria terminalis (BNST) and the amygdala with its basolateral (LB), central (CM), and superficial (SF) nucleus are crucial structures for on organism to respond adaptively to environmental threats (Pessoa, 2011). It has become clear in recent years that complex neuronal interactions within the BNST‐amygdala circuit mediate phasic and sustained fear responses (for review, see Davis, Walker, Miles, & Grillon, 2010). Much progress has been made to understand the specific micro‐circuits involved in these different behavioral and physiological responses going along with phasic and sustained fear (Duvarci & Pare, 2014; Fadok, Markovic, Tovote, & Lüthi, 2018; Krabbe, Gründemann, & Lüthi, 2017; Tovote, Fadok, & Lüthi, 2015). The repertoire of methods to study and manipulate neuronal circuits mostly consisted of lesioning specific regions as well as electrical stimulation and more recently pharmacological and optogenetic manipulations. Although these methods enable more precise stimulation with a high spatial and temporal resolution, experiments using these methods were done in animals and only allowed a limited transfer to human neuronal circuits.

Nevertheless, a variety of studies measuring fMRI BOLD activations on human subjects investigated the role of the BNST and amygdala in specific experimental settings, mostly designed to induce specific or anticipated fear or anxiety responses (for an overview, see Fox & Shackman, 2019; Shackman & Fox, 2016). However, this type of study did not enable insights into the specific connectivity patterns and dynamical interactions between these regions. While studies exist that assessed the connectivity of amygdala (Di, Huang, & Biswal, 2016) and BNST (Brinkmann et al., 2017; Herrmann et al., 2016) with other brain regions during experimental manipulations as well as during the resting‐state (Gorka, Torrisi, Shackman, Grillon, & Ernst, 2018; Kerestes, Chase, Phillips, Ladouceur, & Eickhoff, 2017; Rabellino et al., 2018; Roy et al., 2009; Tillman et al., 2018; Torrisi et al., 2015; Weis, Huggins, Bennett, Parisi, & Larson, 2019), these studies were based on symmetrical measures of statistical dependencies, that is, functional connectivity (FC). Although the results of these studies point towards a positive interconnectedness between BNST and amygdala nuclei, the calculation of FC does not enable inference about the true, that is, effective connectivity (EC) between regions and their dynamical interactions (Friston, 2011). For example, two regions can show substantial FC despite the absence of any true connection, just because of a common input from a third region (Friston, 2011). Moreover, since correlations depend on the level of observation noise, changes in FC arise by merely varying the signal‐to‐noise ratio, for example, by increasing the number of time points or the sample size (Friston, 2011). Moreover, changes in FC also arise by changing the amplitudes of neuronal fluctuations (Friston, 2011). Additionally, previous studies investigated the FC of amygdala and BNST without taking into account the activity of other regions (i.e., no partial correlations). Because of the lack of FC measures to capture dependencies and interactions between regions, dynamic causal modeling (DCM) was developed. DCM models a set of brain regions as differential equations taking into account neuronal interactions between regions and also allows to take the effect of external stimuli into account and can be used for the resting‐state as well (Friston, Harrison, & Penny, 2003; Friston, Kahan, Biswal, & Razi, 2014). In comparison to electrical stimulation, pharmacological or optogenetic manipulations, which are mostly limited to animals, DCM is an approach to understand complex network interactions and to study alterations in humans by means of fMRI and also EEG and MEG (Kiebel, Garrido, Moran, Chen, & Friston, 2009).

In a previous study, we investigated the resting‐state fMRI EC by means of DCM between the BNST and amygdala nuclei in a sample of 391 subjects and reported a pattern of negative EC from the amygdala nuclei to the BNST and positive EC from the BNST to the amygdala, shaping partially anti‐correlated and out‐of‐phase dynamics (Hofmann & Straube, 2019). This pattern was found to be independent of the hemisphere. However, up until now, a reproduction of these results has not been done. Moreover, given the well‐researched relation between BNST, amygdala and anxiety/fear (e.g., Calhoon & Tye, 2015; Davis et al., 2010; Fox & Shackman, 2019), an investigation of the association of EC within the BNST‐amygdala circuit with measures of anxiety might provide a deeper insight into how interregional resting‐state dynamics contributes in shaping specific cognitive and behavioral manifestations of anxiety/fear in humans. So far, there has not been an investigation into the relation between resting‐state EC and anxiety, but other fMRI studies in humans point towards a possible association. For example, a recent study from our lab found a positive correlation between BNST‐amygdala connectivity and trait‐anxiety. The connectivity was measured with psychophysiological interactions comparing the difference in connectivity for aversive versus neutral images. Furthermore, several other studies found that phasic and sustained fear responses in BNST and amygdala in reaction to the presentation of aversive sounds are altered in a variety of anxiety disorders (Brinkmann et al., 2017; Brinkmann, Buff, Feldker, et al., 2017; Buff et al., 2017).

To fill this gap and to build on our previous research, the following study aimed to reproduce our earlier results in three additional fMRI resting‐state samples, including a 7 T sample and investigated the association of the EC parameters with two self‐report anxiety measures.

## 2 METHODS

In the following sections, we will describe the methods and samples in more detail.

### 2.1 Resting‐state samples

For this study, four freely available samples were used. An overview of the characteristics of each sample and the used anxiety measures can be found in Table 1.

**TABLE 1:** Sample characteristics for the different samples

	HCP 3 T	HCP 7 T	NKI	MS	Overall
N (male / female)	384 (172/212)	177 (72/105)	137 (81/56)	121 (38/83)	819 (363/456)
Age in years (M ± *SD*)	28.71 ± 3.67	29.41 ± 3.35	39.58 ± 17.17	25.81 ± 6.02	30.25 ± 9.05
Age (minimum – Maximum)	22–36	22–36	18–85	18–60	18–85
STAI‐T (M ± *SD*)	N/A	N/A	33.42 ± 10.45	30.45 ± 5.86	N/A
DSM anxiety (M ± *SD*)	5.56 ± 5.16	3.54 ± 2.25	N/A	N/A	N/A

*Note:* See section “Description of anxiety questionnaires” for more details. The most right column shows the sample characteristics of all samples subsumed.

Abbreviations: DSM anxiety, anxiety scale of the achenbach adult self‐report; M, mean; N/A, not available; STAI‐T, trait scale of the state–trait anxiety inventory.

#### 2.1.1 Human connectome project 3 and 7 Tesla sample

From the final 1,206 Human Connectome Project 3 T sample (HCP 3 T), we selected all subjects that were unrelated (*n* = 457 in total) and excluded all subjects that had incomplete resting‐state data and missing age, sex, or anxiety questionnaire data. The sample thus consisted of 384 unrelated healthy subjects (Table 1). Note that this is the same sample that was used in our previous study (Hofmann & Straube, 2019), except that we had to exclude seven subjects due to missing anxiety questionnaire data. The HCP 7 T sample (HCP 7 T) consisted of 184 unrelated subjects, from which we excluded subjects that had incomplete resting‐state data and missing age, sex, or anxiety questionnaire data. In total, we included 177 subjects (Table 1). We did not exclude any additional subjects since the HCP samples already consisted of subjects that were free of neurodevelopmental, neuropsychiatric and neurologic disorders and met a series of further inclusion criteria (Van Essen et al., 2013). The data are publicly available at the HCP online database (https://www.humanconnectome.org). Information on the age of the participants was obtained after acceptance of the open and restricted access agreements put forward by the Consortium of the human connectome project. Subject recruitment procedures and informed consent forms were approved by the Washington University institutional review board. All data presented in this paper is not identifiable. Note that 108 of 177 subjects of the 7 T sample, were also included in the 3 T sample since some subjects both had 7 T and 3 T scanning sessions.

#### 2.1.2 Nathan Kline Institute sample

The Nathan Kline Institute (NKI)/Rockland sample was acquired from the Functional Connectomes Project (FCP) website (http://fcon_1000.projects.nitrc.org/indi/pro/nki.html). Details on recruitment and sampling strategy of the sample can be found on the website (http://fcon_1000.projects.nitrc.org/indi/enhanced/recruit.html). The available sample comprised 204 healthy subjects (for details see Nooner et al., 2012) from which we excluded subjects with missing age, sex and anxiety questionnaire information. In total, 137 subjects were used.

#### 2.1.3 Muenster sample

The Muenster sample (MS) consisted of 121 healthy subjects recruited at our own lab. Inclusion criteria for all participants were German as a native language, normal or corrected‐to‐normal vision, and right‐handed. Exclusion criteria were psychiatric medication, neurological disorders, presence, or history of psychotic or bipolar disorder, drug dependence, or abuse within the last 10 years, suicidal ideations, and fMRI contraindications. All subjects gave written informed consent. The study conformed to the Declaration of Helsinki and was approved by the ethics committee of the University of Muenster.

### 2.2 Data acquisition

A summary of the scanning parameters for each sample can be found in Table 2.

**TABLE 2:** Description of scanning parameters for the different samples

	HCP 3 T	HCP 7 T	NKI	MS
Sequence	Gradient‐echo EPI	Gradient‐echo EPI	Gradient‐echo EPI	Gradient‐echo EPI
TR	720 ms	1,000 ms	2,500 ms	2080 ms
TE	30.1 ms	22.2 ms	30 ms	30 ms
Flip angle	52°	45°	80°	90°
FOV	208 × 180 mm	208 × 208 mm	216 × 216	208 × 208
Matrix	104 × 90	130 × 130	72 × 72	92 × 92
Slice thickness	2.0 mm	1.6 mm	3 mm	3 mm
Number of slices	72	85	38	36
Voxel size	2 × 2 × 2 mm	1.6 × 1.6 × 1.6 mm	3 × 3 × 3 mm	2.3 × 2.3 × 3 mm
Multiband factor	8	5		
Image acceleration factor (iPAT)	2	2	None	mSENSE
Partial Fourier (pF) sampling	7/8	7/8	Off	Off
Echo spacing	0.64	0.64	0.51 ms	0.5 ms
BW	1924 Hz/Px	1924 Hz/Px	2,240 Hz/Px	2,470 Hz/Px
Runs	4 (only 2 runs were used)	4 (only 2 runs were used)	1	1
Volumes	900	900	260	202
Duration	~16 min	~16 min	~10 min	~7 min
Eyes	Open/fixated	Open/fixated	Open	Closed

#### 2.2.1 Data acquisition HCP 3 T sample

The data was acquired on a 3 T Skyra Siemens system using a 32‐channel head coil, a customized SC72 gradient insert (100 mT/m) and a customized body transmit coil. The anatomical images were acquired with a high resolution (0.7 mm isotropic) T1‐weighted magnetization prepared rapid gradient echo (3D‐MPRAGE) sequence (TR 2400 ms, TE 2.14 ms, flip angle: 8°. FOV 224 × 224) and the functional images were acquired using a multi‐band gradient‐echo EPI sequence (TR 720 ms, TE 33.1 ms, resolution 2 mm isotropic, 72 oblique axial slices, flip angle 52°, FOV 208 × 180 mm, matrix 104 × 90, echo spacing 0.58 ms, 1,200 images per rsfMRI run). Specifically, rsfMRI data were acquired in four runs of ~15 min each, two runs in one session and two in another session with eyes open with a relaxed fixation on a projected bright cross‐hair on a dark background. Within each session, oblique axial acquisition alternated between phase encoding in a posterior‐to‐anterior direction in one run and phase encoding in an anterior‐to‐posterior direction in the other run.

#### 2.2.2 Data acquisition HCP 7 T sample

No structural scans were acquired on the 7 T scanner and the data from the 3 T sample was used. The functional images were acquired on a Siemens Magnetom 7 T MR Scanner with a Nova32 32‐channel Siemens head coil using a multi‐band gradient‐echo EPI sequence (TR 1000 ms, TE 22.2 ms, resolution 1.6 mm isotropic, 85 oblique axial slices, flip angle 45°, FOV 208 × 208, matrix 130 × 130, echo spacing 0.64 ms, 900 images per rsfMRI run). Specifically, rsfMRI data were acquired in four runs of ~16 min each, with eyes open with relaxed fixation on a projected bright cross‐hair on a dark background. Within each session, oblique axial acquisition alternated between phase encoding in a posterior‐to‐anterior direction in one run and phase encoding in an anterior‐to‐posterior direction in the other run.

#### 2.2.3 Data acquisition NKI sample

The data acquisition was carried out with a Siemens 3 T Magnetom TrioTrim syngo MR B15. Structural images were acquired using a sagittal magnetization T1‐weighted (MPRAGE) sequence (TR = 2,500 ms, TE = 3.5 ms, voxel size = 1 mm isotropic, flip angle = 8°) with 192 slices. The functional resting‐state images were collected by using 260 volumes of a gradient‐echo planar sequence sensitive to BOLD contrast (TR = 2,500 ms, TE = 30 ms, matrix = 72 × 72 voxel, FOV = 216 × 216 mm, flip angle = 80°). Each volume consisted of 38 axial slices (thickness = 3 mm, gap = 0.33 mm, voxel size = 3 × 3 × 3 mm). A shimming field was applied before functional imaging to minimize magnetic field inhomogeneity. The whole resting‐state sequence lasted 10 min. Subjects were instructed to keep their eyes open.

#### 2.2.4 Data acquisition MS sample

Data acquisition was carried out with a Siemens 3 T Magnetom PRISMA and a 20‐channel Siemens head coil. Structural images were acquired using a sagittal magnetization T1‐weighted (MPRAGE) sequence (TR = 2,130 ms, TE = 2.28 ms, voxel size = 1 mm isotropic, flip angle = 8°) with 192 slices. The functional resting‐state images were collected by using 202 volumes of a gradient‐echo planar sequence sensitive to BOLD contrast (TR = 2080 ms, TE = 30 ms, matrix = 92 × 92 voxel, FOV = 208 × 208 mm, flip angle = 90°). Each volume consisted of 36 axial slices (thickness = 3 mm, gap = 0.3 mm, voxel size = 2.3 × 2.3 × 3 mm). A shimming field was applied before functional imaging to minimize magnetic field inhomogeneity. The whole resting‐state sequence lasted 7 min. Subjects were instructed to keep their eyes closed, not to fall asleep and “*let their thoughts flow*.”

### 2.3 Data preprocessing

#### 2.3.1 Data preprocessing of HCP 3 T sample

The data from the HCP 3 T consisted of the extended ICA‐FIX denoised resting‐state fMRI data sets from the first two sessions. All preprocessing of HCP data was carried out in FSL and specifically designed for the HCP acquisition protocols. For a detailed description of the HCP preprocessing methods, please see Glasser et al. (2013) and Smith et al. (2013). Briefly, the minimal preprocessing pipeline for the fMRI data consisted of gradient distortion correction to remove spatial distortions, followed by realignment of volumes to compensate for subject motion, coregistration of the fMRI data to the structural image, nonlinear registration to MNI space, intensity normalization to a mean of 10,000, bias field removal and masking of the data with a final brain mask. No overt volume smoothing was applied, and special care was taken to minimize smoothing from interpolation. After application of the minimal preprocessing pipeline, further processing was done for the resting‐state data. For this, the data was cleaned of structured noise by combining independent component analysis (ICA) with the automated component classifier tool FIX (FMRIB's ICA‐based X‐noisifier) (Griffanti et al., 2014; Salimi‐Khorshidi et al., 2014). FIX classifies the ICA‐detected components into “good” and “bad” (artifactual) and has been specifically trained on HCP data. The artifactual components were then removed in a non‐aggressive manner, that is, removing only the unique variance associated with each component. This approach avoids removing potential variance of interest (Smith et al., 2013). Finally, head motion time series were regressed out by using a 24 confound time series containing the 6 rigid‐body parameter time series, their temporal derivatives as well as the resulting 12 regressors squared). In order to profit from the high quality of the HCP data, we did not apply any additional preprocessing steps except for filtering low‐frequency scanner drifts with a high‐pass filter cutoff of 200 s. For the subsequent DCM analysis, no additional filtering in the frequency range between 0.01 and 0.1 Hz was applied since the spectral DCM based analysis explicitly models the cross‐spectral density within this frequency range.

The data for the first two runs was concatenated to obtain a data set consisting of 2,400 volumes in total. Before concatenation, we first mean‐centered and then variance normalized the data by dividing by the temporal *SD* of the unstructured noise. The unstructured noise temporal *SD* was obtained by first regressing out all signal components of the time series at each voxel, leaving only the noise components, and then calculating the temporal *SD* of the time series at each voxel. By this, it was ensured that the unstructured noise magnitude was distributed equally across the brain for each subject. The signal components were obtained by independent component analysis and already included in the ICA‐FIX extended data set.

#### 2.3.2 Data preprocessing of HCP 7 T sample

The preprocessing pipeline for the HCP 7 T was mostly equal to the HCP 3 T pipeline described in the previous section. However, a different method for the bias field correction of the fMRI scans was used. In this sample, the first two sessions were used for each subject (1800 volumes in total).

#### 2.3.3 Data preprocessing of MS and NKI sample

All preprocessing was carried out using the *Data Processing Assistant for Resting‐State fMRI* (DPARSF, (Yan, Craddock, Zuo, Zang, & Milham, 2013)), which is based on *SPM12* and part of the more comprehensive toolbox for *Data Processing & Analysis of Brain Imaging* (DPABI V3.1_180801, (Yan, Di Wang, Zuo, & Zang, 2016)). The first 5 data volumes were discarded due to spin saturation effects. The remaining volumes were slice‐time corrected and realigned using a six‐parameter (rigid body) linear transformation. The anatomical and functional images were coregistered and then segmented into gray matter (GM), white matter (WM) and cerebrospinal fluid (CSF). In order to consider possible confounding effects of head motion, the Friston 24‐parameter (i.e., six head motion parameters, six head motions parameters one point in time before, and the 12 corresponding squared items) model was used to regress out head motion effects from the realigned data (Friston, Williams, Howard, Frackowiak, & Turner, 1996). WM and CSF signals were regressed out using the *CompCor* method (Behzadi, Restom, Liau, & Liu, 2007). In addition, linear and quadratic trends were included as nuisance regressors because of low‐frequency drifts of the BOLD signal. No temporal filtering (0.01–0.1 Hz) was applied. The data was then nonlinearly normalized to MNI152 standard space with DARTEL (Ashburner, 2007) and resampled to 2 mm isotropic voxels. No spatial smoothing was applied.

#### 2.3.4 Regions of interest selection

**FIGURE 1:**
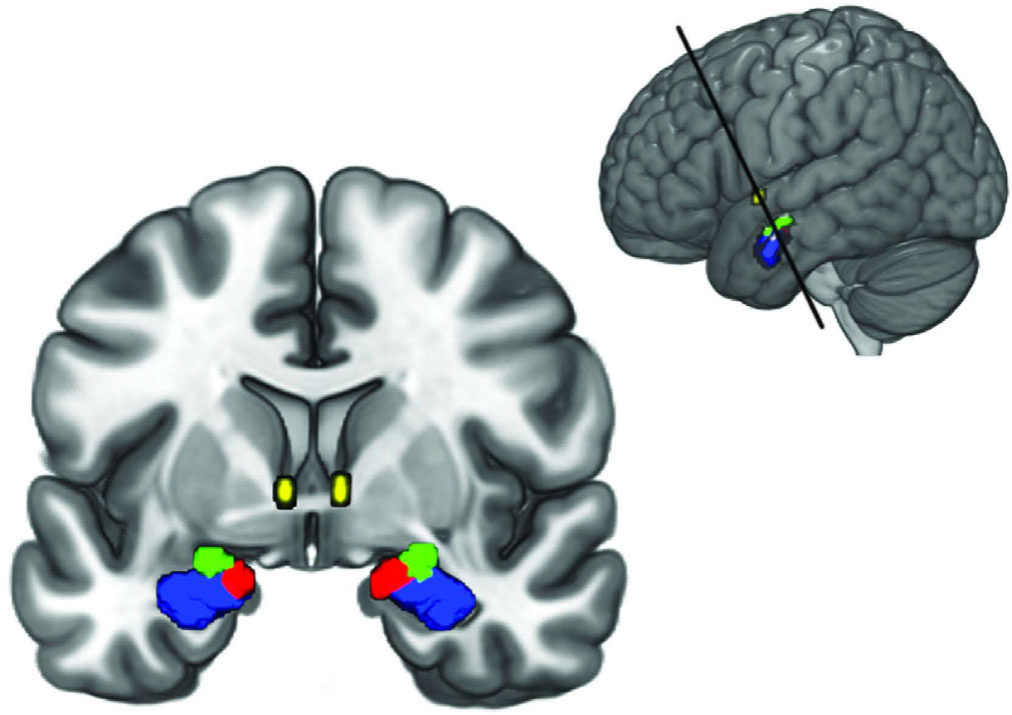
Amygdala nuclei and BNST regions of interest used in this study. The LB is shown in blue, the CM in green, the SF in red and the BNST in yellow. The top right inlay shows the section

For the subsequent DCM analysis, masks of amygdala nuclei were obtained from the Anatomy Toolbox (AT) version 2.2c available for SPM (Eickhoff et al., 2005). The masks consisted of the maximum probability maps (MPM) of the LB, CM, and SF (Amunts et al., 2005; Eickhoff et al., 2005). The mask for the BNST was obtained from the probabilistic atlas developed by Torrisi et al. (2015). For the BNST mask, we included all voxels with at least 80% probability of being located within the BNST. Different masks were created for the left and right hemispheres separately as well as for both hemispheres together. See Figure 1 for an anatomical representation of the location of the regions.

#### 2.3.5 Regions of interest extraction

Region of interest (ROI) time series extraction for the later DCM analysis was performed using the volume‐of‐interest tool included in SPM12 (v7219). ROI time series were obtained by selection of the first principal component, that is, the component that explained the most variance after calculation of a principal component analysis that included the time series of all voxels within an ROI. Before extraction, we ensured that all subjects had sufficient EPI coverage of the ROIs, which was the case for all subjects in our sample.

### 2.4 DCM

All analysis was done using SPM12 (v7219) in MATLAB 2017b. After extraction of the ROI time series, we calculated a one‐state spectral DCM, specifying a model of EC in which all ROIs were connected. Spectral DCM is a variant of DCM that is suited for the estimation of EC in resting‐state fMRI data and uses a neuronally plausible model of coupled neuronal states to generate complex cross‐spectra (Friston et al., 2014). In other words, the generative model is identical to the deterministic DCM used in fMRI time series analysis (Friston et al., 2003) but is used to predict the sample cross‐spectra as opposed to the time series themselves. In comparison to previous approaches based on stochastic differential equations (stochastic DCM), this variant enables faster, more accurate and less computationally intensive estimation of EC (Razi et al., 2017; Razi, Kahan, Rees, & Friston, 2014). The DCM used here consists of a system of random differential equations that model neuronal interactions of the form:

(1) $$\dot{x}\left(t\right)=A\;x\left(t\right)+v\left(t\right)$$

Where **x**(*t*) = [*x*_1_(*t*), …, *x*_4_(*t*)]^T^ is a column vector of hidden neuronal states for the four regions BNST, LB, CM, and SF, whose activity depends on the other regions and endogenous fluctuations modeled by **v**(*t*). The hidden states are abstract representations of neuronal activity that correspond to the amplitude of macroscopic variables, which summarize the dynamics of large neuronal populations. The endogenous fluctuations are generated from an AR (1) process with an autoregression coefficient of one half. **A** is a four by four matrix with the unknown coupling coefficients between regions, that is, the effective connectivity parameters in units of Hz to be estimated given the data. The resulting output values then serve as input to equations that generate the hemodynamic responses of each ROI. The equations that generate the hemodynamic response are not shown here. For details, see Friston et al. (2003). This DCM is then fitted to the cross‐spectra of the extracted ROI time courses of each subject.

### 2.5 Parametric empirical Bayes for group DCM

After fitting each subject's DCM for each sample to their fMRI data, we ran a second‐level analysis in order to estimate the group mean of each sample and the effects of the covariates age, sex and anxiety for each connectivity parameter of the model. This analysis was based on the recently developed parametric empirical Bayes (PEB) method that models connectivity at the group level by means of a hierarchical Bayesian model (Friston et al., 2016). The subject‐specific connectivity estimates (consisting of the expected values and covariances) are taken to the group‐level by fitting a Bayesian GLM to the data. Other than tests based on classical statistics, PEB uses the full posterior density over the connectivity parameters from each subject's DCM to inform results on the group‐level. It thus takes into account both the expected strength of the connection and its uncertainty (posterior covariance). In other words, subjects are weighted by the precision of their estimates, such that subjects with noisy estimates contribute less to the group result (Zeidman et al., 2019). This analysis was repeated for three sets of ROIs, the bilateral ROIs, as well as for the left and right hemispheric ROIs. Since our previous study revealed that a fully connected BNST‐amygdala model is most plausible (Hofmann & Straube, 2019), we did not choose to perform a Bayesian model reduction.

### 2.6 Description of anxiety questionnaires

#### 2.6.1 DSM_anxiety

For the HCP samples, the measure of anxiety used was part of the Achenbach Adult Self‐Report (ASR) Scale. The ASR is a 126‐item self‐report scale for adults, which measures aspects of adaptive functioning and problems (Rescorla & Achenbach, 2004). The anxiety scale ist based on the operationalization of anxiety in the Diagnostic and Statistical Manual of Mental Disorders (DSM). That is, experts rated the consistency of the items with the DSM diagnostic categories for anxiety. The items which were rated as most consistent by a majority of experts were then included in the anxiety scale (Rescorla & Achenbach, 2004).

#### 2.6.2 STAIT‐T

For the MS and NKI samples, the state–trait anxiety inventory (STAI) was available (Spielberger, Gorsuch, & Luschene, 1970). The scale measures both state and trait anxiety. Here we used only the scale for trait anxiety. For the MS sample, the German version of the STAI was used (Laux, Glanzmann, Schaffner, & Spielberger, 1981).

## 3 RESULTS

**FIGURE 2:**
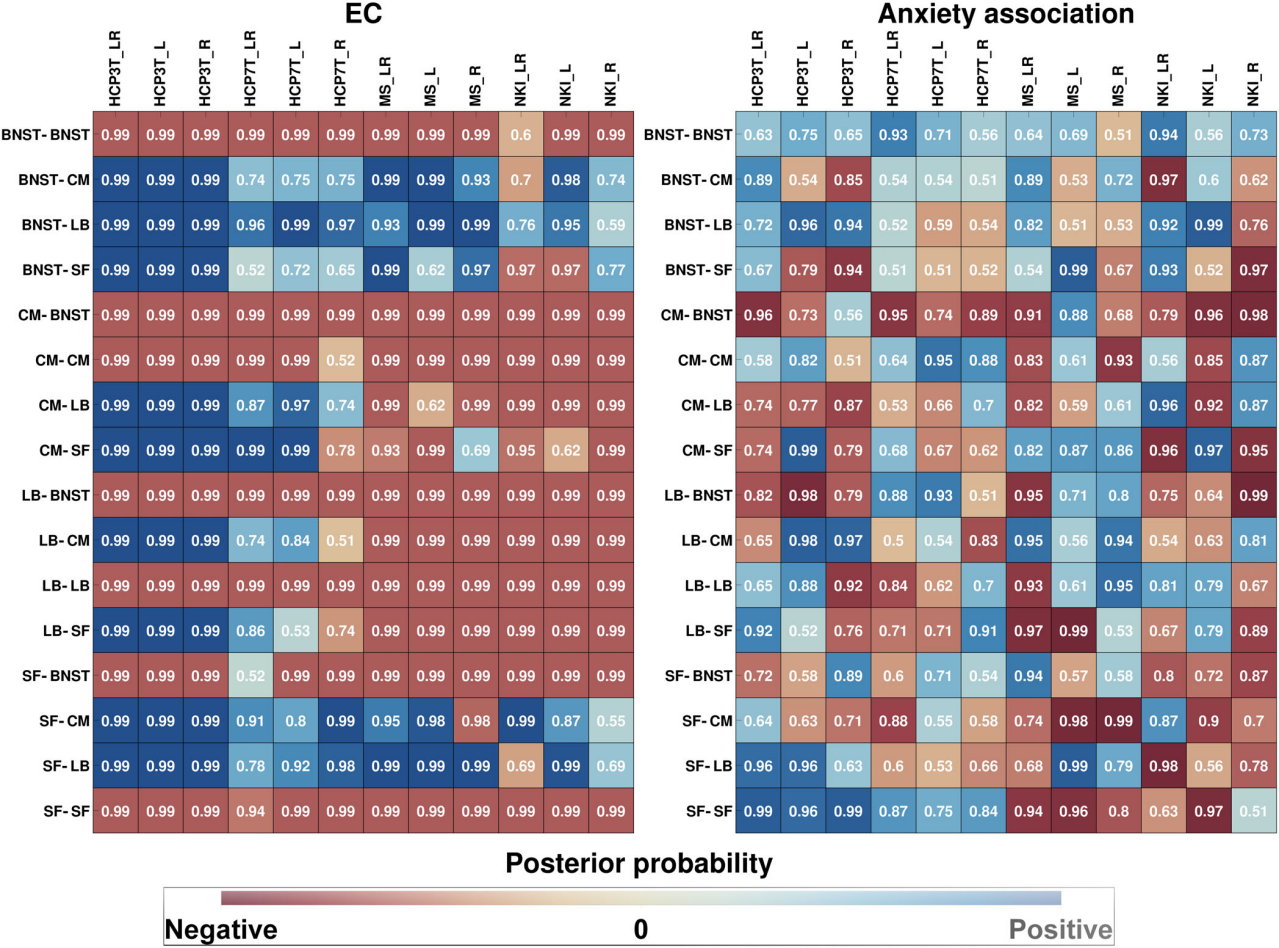
Similarities between sites for each EC parameter (left) and its association with anxiety (right). Site names are shown as column headers, connections between regions are shown as row headers. The numbers in the boxes show the posterior probability. The blue color tones represent the posterior probabilities of positive EC values and positive anxiety associations, respectively, while the red color tones represent the posterior probabilities of negative EC values and negative anxiety associations, respectively. Note that the suffixes L, R, and LR in the column headers indicate the type of ROIs that were used (L: left hemispheric, R: right hemispheric, LR: both hemispheres combined)

**FIGURE 3:**
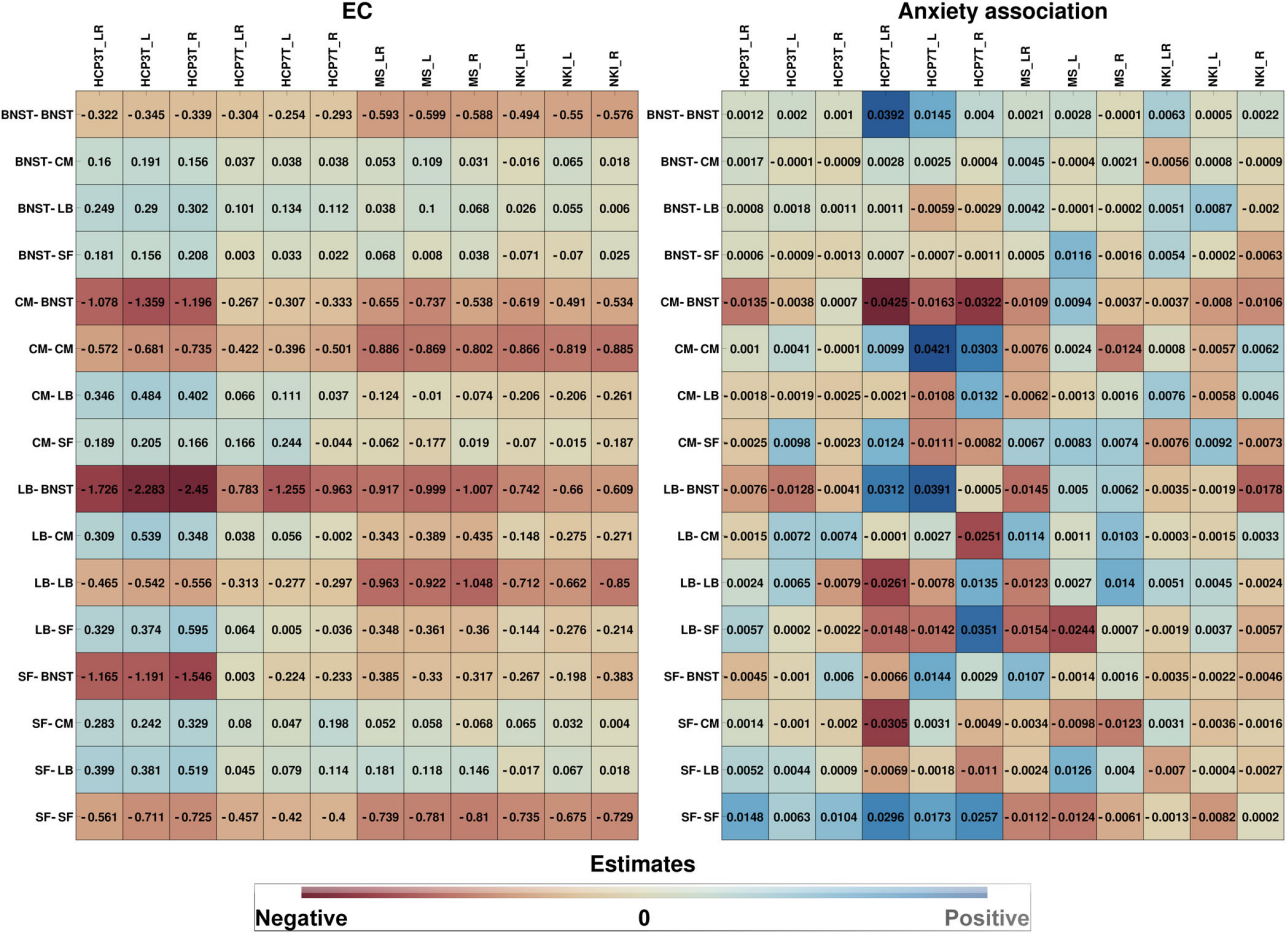
EC estimates (left) and association strength with anxiety (right). Site names are shown as column headers, connections between regions are shown as row headers. The blue color tones represent positive EC estimates and positive anxiety associations, respectively, while the red color tones represent negative EC estimates and negative anxiety associations, respectively. Note that the suffixes L, R, and LR in the column headers indicate the type of ROIs that were used (L: left hemispheric, R: right hemispheric, LR: both hemispheres combined)

In the following, we present the results of the DCM estimation for the different samples for the bilateral as well as left and right hemispheric ROI selection. Figure 3 shows the estimated EC values and the strength of association of the EC with anxiety. Figure 2 shows the posterior probability (Pp) of these estimates. In Figures S1 and S2 and the supplementary excel tables, the reader will find a detailed overview of the DCM results for each site separately and the exact EC parameter values with 95%‐credible intervals.

### 3.1 EC

As can be seen from Figure 2, there was a relatively high coherence of EC parameter values between the different sites and hemispheres for some connections, while for others, it was more heterogenic. The CM‐BNST and LB‐BNST negative EC can be found in all sites irrespective of the ROI selection with high Pp. The SF‐BNST EC was also mostly negative, except in the HCP7T_LR sample where it was positive with a Pp of 0.52 of being unequal to zero. Moreover, Figure 3 indicates that the EC strengths for these connections were largest for the HCP 3 T sample.

The positive EC from the BNST to the amygdala nuclei (BNST‐CM, BNST‐LB, and BNST‐SF) was also mostly coherent over samples but was more heterogenic for the BNST‐CM and BNST‐SF. That is, in the NKI_LR sample, the BNST‐CM was negative with a Pp of 0.7 and the BNST‐SF EC was negative with a Pp of 0.97 in the NKI_LR as well as in the NKI_L sample. Generally, the EC strengths for these connections were weaker as compared with the negative EC from the amygdala to the BNST.

As can also be seen, the within‐amygdala EC (CM‐LB, CM‐SF, LB‐CM, LB‐SF, SF‐CM, SF‐LB) was mostly incoherent over the different samples and ROI selections. An exception was the SF‐CM and SF‐LB EC, which was mainly positive, except in the MS_R and NKI_LR sample, where it was negative with a Pp of 0.98 and 0.69.

### 3.2 Association of EC with self‐reported anxiety

For the results of the analysis of the association between EC and anxiety, it is important to note, that the anxiety measures used are different between the HCP samples and the MS and NKI samples, each scale capturing a different aspect of anxiety. We will restrict our report of the results to the commonalities over the samples since a connection that is associated with both anxiety measures, captures common aspects of the different anxiety conceptualizations.

As can be seen in the right subfigure of Figure 2, the only connections that were largely coherent between samples, were the BNST‐BNST and the CM‐BNST connections. In other words, the BNST‐BNST connection was positively associated with anxiety in all samples, except the MS_R sample, with a maximum Pp of 0.94 in the NKI_LR sample and a minimum Pp of 0.56 in the NKI_L sample. The CM‐BNST connection was negatively associated with anxiety, except in the HCP3T_R and MS_L sample, with a maximum Pp of 0.99 in the NKI_R sample and a minimum Pp of 0.74 in the HCP7T_L sample.

## 4 DISCUSSION

Our investigation of the commonalities of the resting‐state EC within the BNST‐amygdala circuit in four different data sets, suggests a partially reproducible pattern of EC with only minor association strengths of some EC parameters with measures of anxiety.

To summarize, we found a robust negative EC from the amygdala (LB and CM) to the BNST. This pattern was present in all sites and in both the bilateral (left and right hemisphere combined) and unilateral (left and right hemisphere separated) DCMs. Moreover, we found a negative SF‐BNST EC in all but the HCP7T_LR DCMs. These findings suggest a mostly coherent pattern of negative EC between amygdala nuclei and the BNST independent of the hemisphere. However, there were large variations in the relative EC strengths between the samples, most likely due to the different sample sizes.

The positive EC was less coherent between sites and hemispheres, only showing a consistent influence from the BNST to the LB in all samples. The other connections (BNST‐CM, BNST‐SF) could not be reliably reproduced. However, the positive BNST‐CM EC was present in all DCMs except the NKI_LR sample, and the BNST‐SF EC was present in all but the NKI_LR and NKI_L sample. It is not clear, at the moment, why the NKI sample shows these discrepancies. However, despite these discrepancies, the results indicate a tendency toward a positive influence from the BNST to the amygdala.

The within‐amygdala EC could not be reliably reproduced, showing large differences between the samples. However, the SF‐CM EC was found to be positive in all, but the MS_R sample and the SF‐LB EC was found to be positive in all but the NKI_LR sample. This indicates that most of the samples showed a positive influence from the SF to the CM and LB.

As has been discussed in our previous work (Hofmann & Straube, 2019), the pattern of EC within the BNST‐amygdala circuit points towards a strong negative influence from the amygdala to the BNST, while the BNST exerts a smaller positive influence on the amygdala nuclei. As has also been pointed out in our previous work, this pattern of EC still seems to shape a partially antagonistic “information flow” between BNST and amygdala. In other words, activation of the amygdala leads to a strong and fast inhibition of the BNST, in turn generating slower inhibitory feedback to the amygdala through the smaller positive EC arriving from the BNST. This downregulation of the amygdala then upregulates BNST activity, which in turn leads to an upregulation of amygdala activity but on a slower time scale, thus generating a partially desynchronized activity pattern. Therefore, it seems that—in the resting‐state—BNST and amygdala are somewhat functionally dissociated. As of yet, it is not clear what this dissociation means, but one plausible assumption could be that both structures take part in different computational processes, most likely through association with different brain regions. Evidence from FC studies seems to substantiate this assumption partially. That is, the BNST was found to be functionally connected to regions involved in the Default Mode Network, while the CM and LB do not entirely share the same connectivity structure, despite certain overlaps (Gorka et al., 2018; Tillman et al., 2018; Torrisi et al., 2015; Weis et al., 2019). However, as has been pointed out, a direct comparison of EC and FC is not easily possible.

Although it is speculative at the moment how DCM results are related to the activity on a neuronal level, functional dissociations of BNST and amygdala have been observed in phasic and sustained fear responses (Davis et al., 2010). Specifically, Davis et al. (2010) proposed that a fear‐eliciting stimulus rapidly activates the laterobasal amygdala and the medial part of the central amygdala and triggers a phasic fear response. This response is paralleled by activation of the lateral central amygdala that results in a release of corticotropin‐releasing factor into the BNST to produce a more slowly acting sustained fear response. This phasic fear response is then turned off by inhibitory feedback from the BNST, and possibly the lateral central amygdala, to the medial central amygdala. This pattern of neuronal activity also suggests a partially desynchronized activity pattern between BNST and amygdala, with a rapid response of the amygdala, followed by a slower sustained BNST response.

Evidence from fMRI studies using aversive stimuli also points towards a delayed and sustained BOLD activity of the BNST (Alvarez, Chen, Bodurka, Kaplan, & Grillon, 2011; Brinkmann, Buff, Feldker, et al., 2017; Brinkmann, Buff, Neumeister, et al., 2017; Herrmann et al., 2016; Straube, Mentzel, & Miltner, 2007) and also indicates a complex interaction between BNST and amygdala, such that both contribute to shaping phasic and sustained fear responses (for reviews, see Fox & Shackman, 2019; Shackman & Fox, 2016). Our data also seems to support the view of a dynamic interaction between BNST and amygdala and offers an explanation of how this interaction unfolds in terms of EC between these structures. However, although there are some similarities between our results and the model of Davis et al. (2010), our DCMs were explicitly fit to resting‐state data without any aversive external stimuli. This makes a direct comparison difficult. Nevertheless, our results suggest that even in an experimental setting of an absence of fear‐eliciting stimuli, BNST and amygdala seem to show a pattern of dynamical interaction that shapes partially antagonistic activity. This may reflect some form of baseline information processing between these structures, which is subject to alterations by external stimuli.

To gain more insight into the relationship between EC and anxiety, we investigated the association of the EC with two measures of self‐reported anxiety. The results of the common associations of both anxiety measures with the EC showed small effect sizes and high variability between sites, which could also not be reliably reproduced in the different DCMs separated by hemisphere. Although caution should be taken in interpreting these results, two of them deserve mentioning. Firstly, the CM‐BNST connection was present in all DCMs, but the HCP3T_R and MS_L sample (see Figure S2) and was, therefore, more coherent than other connections. Under the assumption that these results reflect some ground truth, this points towards a small negative association between anxiety and the influence of the CM onto the BNST, that is, the higher anxiety the stronger the inhibitory influence from the CM onto the BNST. Secondly, the association of the BNST self‐connection (BNST‐BNST) with anxiety deserves further mentioning. This connection showed a positive association with anxiety in all DCMs but the MS_R sample. It is, therefore, relatively consistent over sites and the two different anxiety measures. Again assuming a true effect, the finding of higher anxiety with less self‐inhibition of the BNST points toward increased BNST baseline activity as a potential contributor to higher anxiety.

These results are consistent with findings from a study at our lab using the same methodology to compare panic disorder patients with healthy controls. The results of this study showed that patients also have less BNST self‐inhibition and increased inhibition from the CM to the BNST compared with controls (Hofmann, Feldker, & Straube, in prep). It, therefore, seems that higher BNST baseline activity, along with stronger inhibitory influence from the CM to the BNST, is related to increased anxiety. In other words, due to the reduction in self‐inhibition of the BNST, its activation decays more slowly, while the increased inhibition by the CM results in even stronger BNST inhibition, which then leads to more inhibitory feedback from the BNST to the amygdala. This is then followed by stronger positive feedback back to the BNST and, therefore, generally higher and more sustained BNST activity. It is plausible that the resulting increase in amygdala and BNST activation may then contribute to intensified and prolonged states of anxiety due to the hyperactivation of downstream targets involved in autonomic, neuroendocrine, and/or behavioral regulation. For example, both the BNST and CM influence hypothalamic–pituitary–adrenal (HPA) axis activity (Choi et al., 2007; Crestani et al., 2013; Forray & Gysling, 2004; Herman, Ostrander, Mueller, & Figueiredo, 2005) and an increase in the activity amplitude of the BNST‐amygdala circuit might result in elevated HPA‐axis activity of subjects with higher anxiety scores. Interestingly, as has been reported by several studies from our lab, exaggerated phasic amygdala and sustained BNST activity in reaction to aversive stimuli seem to be characteristic of many anxiety disorders. For example, female patients with posttraumatic stress disorder as well as panic disorder patients and patients with generalized anxiety disorder showed increased initial phasic amygdala and increased sustained BNST fMRI BOLD responses during the anticipation of aversive versus neutral sounds as compared with controls (Brinkmann, Buff, Feldker, et al., 2017; Brinkmann, Buff, Neumeister, et al., 2017; Buff et al., 2017). Although these similarities do not seem to be purely coincidental, all of these studies did investigate BNST or amygdala task‐related BOLD activation and a direct comparison between resting‐state and task conditions is not easily possible. Nevertheless, our findings might provide insights into how the interaction between BNST and amygdala generates phasic and—possibly prolonged—sustained responses.

Based on our results, certain future directions of research can be formulated. Firstly, it is important to investigate the differences and similarities of EC in different types of disorders, especially anxiety disorders. Moreover, the resting‐state condition is not suited to examine task‐induced changes in EC. The investigation of the changes by specific tasks will provide a deeper understanding of the task‐related alterations of EC within the BNST‐amygdala circuit.

A final point that has to be discussed pertains to the possible reasons for the lack of reproducibility between samples with regard to the within‐amygdala EC. As can be seen from the results (Figures 1, S1), some connections show high variability between samples (CM‐LB, CM‐SF, LB‐CM, and LB‐SF). In particular, the HCP samples mainly showed a positive EC, while the NKI and MS samples showed a negative EC of these connections. This contrast of EC values is interesting, but at the moment, not well understood and needs further investigation. However, the different quality of the data might be a possible explanation of these discrepancies. That is, the amygdala is a region that is highly susceptibility to signal artifacts caused by magnetic field inhomogeneities due to its proximity to air‐filled cranial spaces, especially in the medial part of the amygdala. Although the HCP data quality was high and corrections for magnetic field inhomogeneities were used, it was less optimal for the MS and NKI sample. Moreover, the MR sequences were not specifically designed for measuring signals in artifact susceptible regions. This might have affected the parameter estimation to some degree, thus causing some of the observed discrepancies between the HCP and MS/NKI samples. Future studies focusing on the BNST‐amygdala circuit should use MR sequences specifically designed to measure BNST and amygdala BOLD responses. For example, Khatamian, Golestani, Ragot, and Chen (2016) demonstrated that spin‐echo echo‐planar imaging, as compared with gradient‐echo echo‐planar imaging, shows higher sensitivity, specificity, and inter‐subject reproducibility in regions that are highly susceptible to artifacts, when calculating resting‐state FC. Given the found heterogeneity in the within‐amygdala EC between samples, future studies should be cautious when interpreting the results of an estimation of within amygdala EC (or FC).

## 5 LIMITATIONS

Several limitations of this study have to be mentioned. Firstly, the results of modeling neuronal circuits by a DCM, do not directly allow inferences about certain subpopulations of neurons or specific circuits on the neuronal level. At present, the used DCM model is, therefore, a highly simplified model, that summarizes the neuronal population activity in abstract variables. For example, our results show that the BNST is positively connected to the CM. This result is in contraction to the results from Gungor, Yamamoto, and Pare (2015), showing that projection neurons from the BNST to the CM are predominantly GABAergic in rats and therefore elicit inhibitory responses. Interestingly, recent developments of DCM for fMRI consist of more detailed neural mass models that allow for an assessment of neocortical neuronal populations, including spiny stellate cells, superficial pyramidal cells, inhibitory interneurons, and deep pyramidal cells (Friston et al., 2019). However, since the amygdala and BNST consist of a large variety of neuronal subpopulations (Babaev, Piletti Chatain, & Krueger‐Burg, 2018; Krabbe et al., 2017) future developments of DCM should be specifically designed to match the neuronal environment of these regions and thus provide a much more detailed understanding of the neuronal interactions.

In addition, we did only focus on calculating the EC between BNST and amygdala nuclei. Since the BNST and amygdala have afferents and efferents to a multitude of other brain regions, the EC reported does not take the influence of these other regions into account. Future studies should, therefore, study the influence of other regions on BNST and amygdala activity to gain a more in‐depth understanding of the dynamical interaction between regions and the association of EC with anxiety.

Moreover, our ROI selection did not include further subdivision of BNST and amygdala. The BNST is a heterogeneous structure consisting of several subnuclei and intermingled cell populations (Gungor & Pare, 2016) that are involved in different functions. For example, BNST subnuclei were found to be involved in opposing circuits that mediate anxiogenic and anxiolytic responses (Kim et al., 2013), fear learning (Haufler, Nagy, & Pare, 2013) or differentially regulate the HPA axis (Choi et al., 2007). In addition, we did not distinguish between the centrolateral and centromedial amygdala, two subdivisions that also have been found to play essential differential roles in the mediation of fear and anxiety responses (Ciocchi et al., 2010; Davis et al., 2010; Duvarci & Pare, 2014). The same is true for a separation between the lateral, basal and basomedial nuclei of the basolateral complex of the amygdala, which also have been shown to mediate different aspects of fear learning and fear expression (Duvarci & Pare, 2014). Our results are therefore limited because the signal components used in the DCM comprise a mixture of signals from several subnuclei. However, at the moment, a distinction between BNST subnuclei as well as subnuclei of the central amygdala is not possible due to the limited spatial resolution of fMRI, although a recent work did differentiate subnuclei of the amygdala in greater detail than previously done (Tyszka & Pauli, 2016).

A final limitation that was pointed out by one of our reviewers pertains to the possible effects of early adversity on the BNST‐amygdala circuit EC. Notably, a history of physical abuse has been found to be correlated with stressor‐evoked changes in mean arterial pressure and BOLD activity in the BNST and amygdala (Banihashemi, Sheu, Midei, & Gianaros, 2015) and, therefore, might also affect the resting‐state. Since the effects of physical abuse on EC have not been explicitly addressed in our study, it cannot be ruled out that subjects with a history of physical abuse show altered within BNST‐amygdala EC. However, since the sample sizes in this study were large, representative of the general population and from different sites, the effects of a history of physical abuse in some subjects likely did not induce a strong bias in the estimation of EC within the BNST‐amygdala circuit. Nevertheless, the effects of physical abuse on resting‐state EC within the BNST‐amygdala circuit should be systematically addressed in future studies.

## 6 CONCLUSION

In conclusion, our results point towards strong inhibitory influence from the amygdala to the BNST and positive EC from the BNST to the amygdala, while higher BNST baseline activity and stronger negative CM‐BNST EC is weakly associated with higher anxiety. These results are mostly reproducible over the different samples. Our study, therefore, adds to our previous investigation and enables further insight into the complex neuronal interaction within the BNST‐amygdala circuit and the relation of EC with anxiety. However, much work has yet to be done and the field is faced with trying to bridge a gap between animal studies directly targeting neuronal structures through stimulation, or optogenetic and pharmacological methods, and modeling of BOLD responses in humans using DCM. Research that focuses on the BNST‐amygdala circuit and how certain conditions and stimuli alter the EC between these structures, as well as their dynamical interaction, will likely enable a more in‐depth insight and comparison with results from animal research.

## Supporting information


**Figure 1** Effective connectivity between the bilateral (left column) and unilateral (middle/right column) BNST, LB, CM and SF for each site separately. Values above/below the arrows show the estimated EC parameter values, the posterior probability of the value being unequal to zero (round brackets) and the 95% credible interval (square brackets). Positive values are shown in green and negative values are shown in red. Dashed arrows indicate that the posterior probability of the values is smaller than 0.9. A list of all estimates and their 95% credible intervals can also be found in the supplementary excel Table 1.
**Figure 2**. Strength of anxiety association with EC parameters values for the bilateral (left column) and unilateral (middle/right column) BNST, LB, CM and SF roi selection for each site separately. Values above/below the arrows show the estimated EC parameter values and the posterior probability of the value being unequal to zero (round brackets). Positive associations are shown in green and negative are shown in red. Dashed arrows indicate that the posterior probability of the values is smaller than 0.9. A full list of all estimates and their 95% credible intervals can also be found in the supplementary excel Table 2.

**Table S1**

**Table S2**

## Data Availability

The data that supports the findings of this study are available in the supplementary material of this article.
