# Supplementary material for: Effective connectivity between bed nucleus of the stria terminalis and amygdala: Reproducibility and relation to anxiety
Source: Hum Brain Mapp. 2020 Nov 6;42(3):824–36. doi: 10.1002/hbm.25265 (PMC7814768; doi:10.1002/hbm.25265)
Supplement: Supplementary file 1 — Figure 1 Effective connectivity between the bilateral (left column) and unilateral (middle/right column) BNST, LB, CM and SF for each site separately. Values above/below the arrows show the estimated EC parameter values, the posterior probability of the value being unequal to zero (round brackets) and the 95% credible interval (square brackets). Positive values are shown in green and negative values are shown in red. Dashed arrows indicate that the posterior probability of the values is smaller than 0.9. A list of all estimates and their 95% credible intervals can also be found in the supplementary excel Table 1. Figure 2. Strength of anxiety association with EC parameters values for the bilateral (left column) and unilateral (middle/right column) BNST, LB, CM and SF roi selection for each site separately. Values above/below the arrows show the estimated EC parameter values and the posterior probability of the value being unequal to zero (round brackets). Positive associations are shown in green and negative are shown in red. Dashed arrows indicate that the posterior probability of the values is smaller than 0.9. A full list of all estimates and their 95% credible intervals can also be found in the supplementary excel Table 2. [file HBM-42-824-s001.docx]

# Supplementary Material

## Bilateral and unilateral EC for all sites


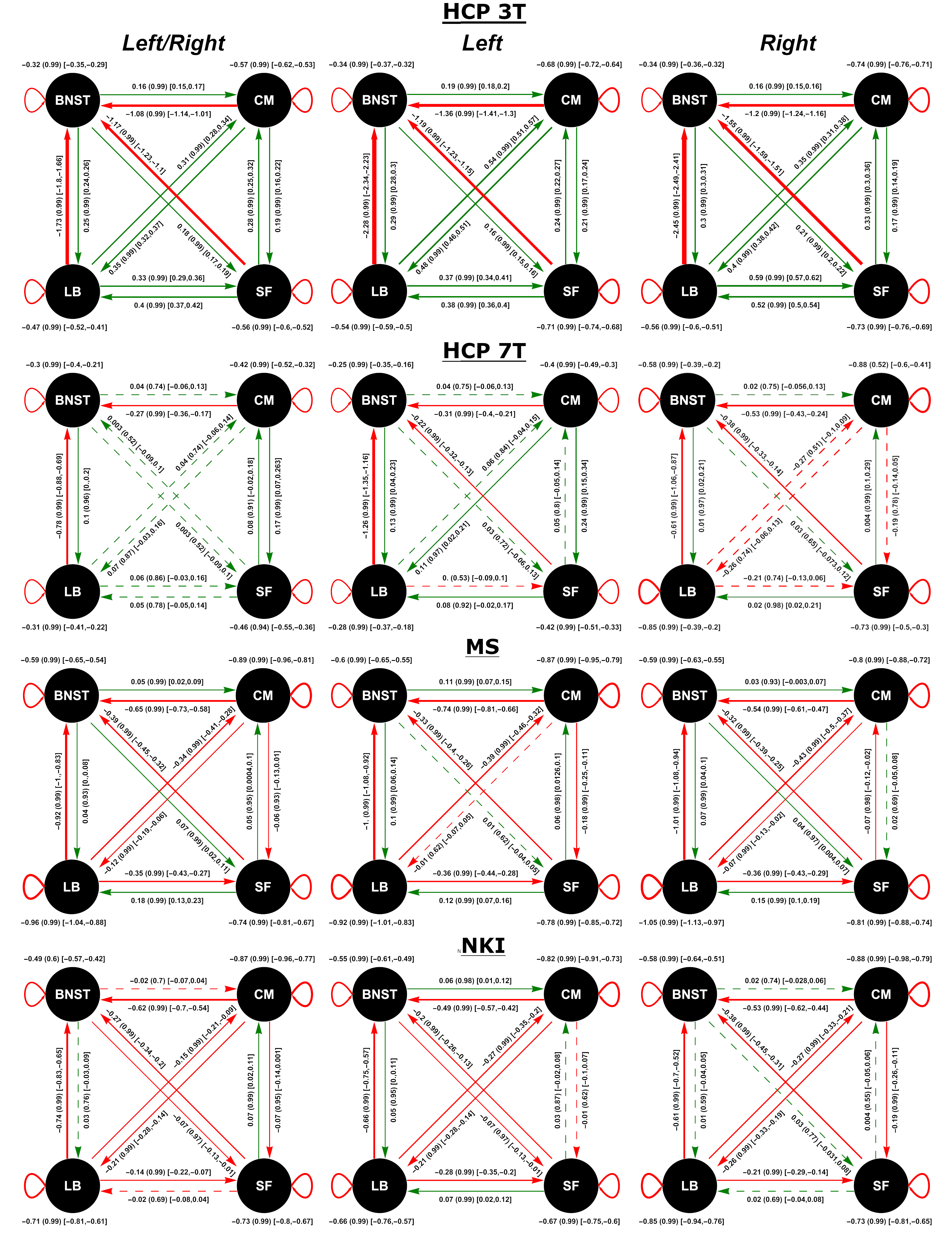


Figure 1. Effective connectivity between the bilateral (left column) and unilateral (middle/right column) BNST, LB, CM and SF for each site separately. Values above/below the arrows show the estimated EC parameter values, the posterior probability of the value being unequal to zero (round brackets) and the 95 % credible interval (square brackets). Positive values are shown in green and negative values are shown in red. Dashed arrows indicate that the posterior probability of the values is smaller than 0.9. A list of all estimates and their 95 % credible intervals can also be found in the supplementary excel table 1.

## Association of EC with anxiety


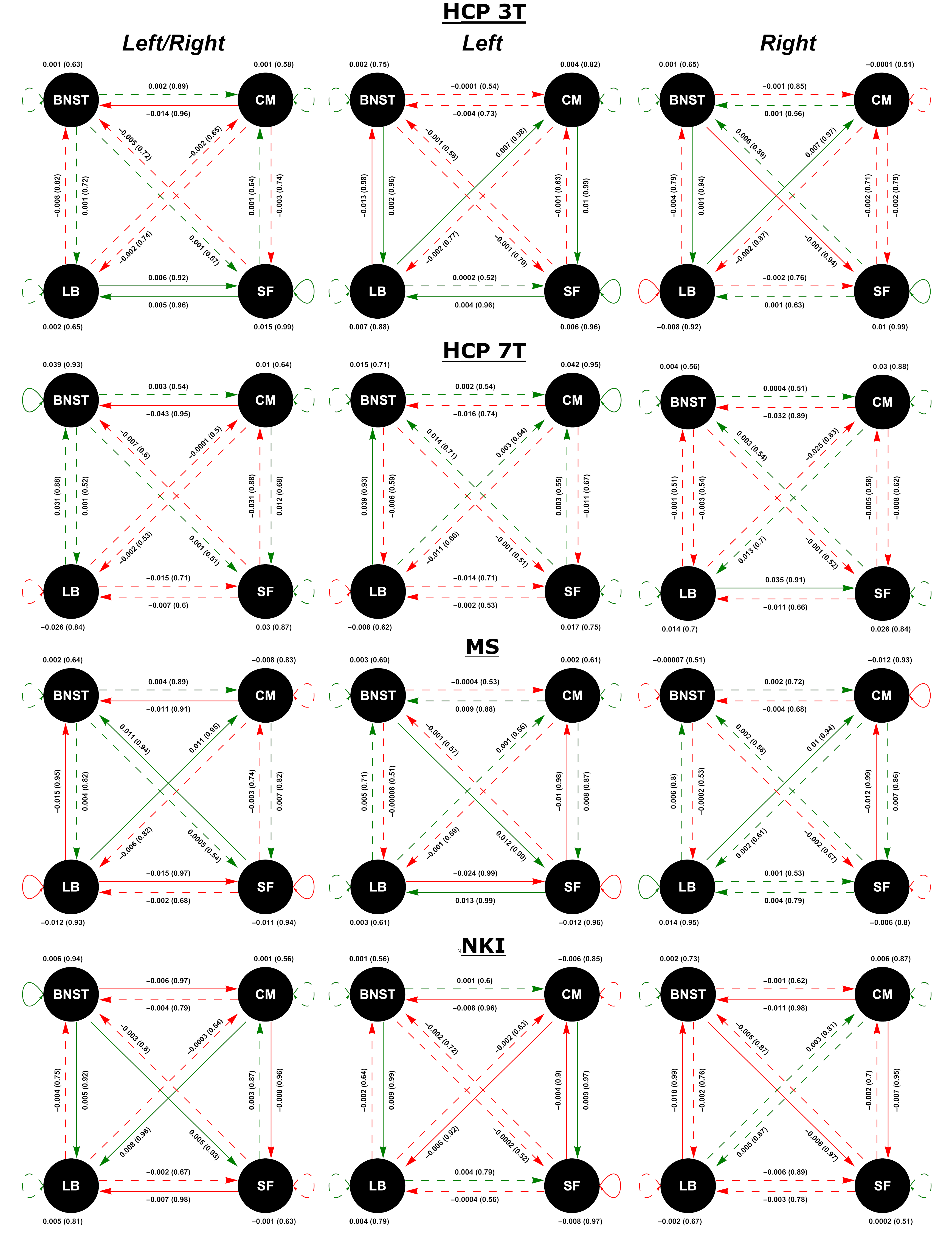


Figure 2. Strength of anxiety association with EC parameters values for the bilateral (left column) and unilateral (middle/right column) BNST, LB, CM and SF roi selection for each site separately. Values above/below the arrows show the estimated EC parameter values and the posterior probability of the value being unequal to zero (round brackets). Positive associations are shown in green and negative are shown in red. Dashed arrows indicate that the posterior probability of the values is smaller than 0.9. A full list of all estimates and their 95 % credible intervals can also be found in the supplementary excel table 2.
